# Heterogeneity in high-risk prostate cancer treated with high-dose radiation therapy and androgen deprivation therapy

**DOI:** 10.1186/s12894-017-0250-2

**Published:** 2017-08-01

**Authors:** Daniel N. Cagney, Mary Dunne, Carmel O’Shea, Marie Finn, Emma Noone, Martina Sheehan, Lesley McDonagh, Lydia O’Sullivan, Pierre Thirion, John Armstrong

**Affiliations:** 1 Department of Radiation Oncology, St. Luke’s Radiation Oncology Network, Highfield Road Rathgar, Dublin, Ireland; 2 Clinical Trials Unit, St. Luke’s Radiation Oncology Network, Dublin, Ireland

## Abstract

**Background:**

Our aim was to assess the heterogeneity of high-risk (HR) prostate cancer managed with high-dose external beam radiotherapy (EBRT) with androgen deprivation therapy (ADT).

**Methods:**

We identified 547 patients who were treated with modern EBRT from 1997 to 2013, of whom 98% received ADT. We analyzed biochemical relapse-free survival (bRFS) and distant metastases-free survival (DMFS).

**Results:**

Median EBRT dose was 74 Gy, and median ADT duration was 8 months. At 5 years, the DMFS was 85%. On multivariate analysis, significant predictors of shorter bRFS were biopsy Gleason score (bGS) of 8 to 10, higher prostate-specific antigen (PSA) level, shorter duration of ADT and lower radiation dose while predictors of shorter DMFS were bGS of 8 to 10, higher PSA level, and lower radiation dose. We identified an unfavorable high-risk (UHR) group of with 2–3 HR factors based on 2015 National Comprehensive Cancer Network (NCCN) criteria and a favorable high-risk (FHR) group, with 1 HR feature. Comparing very-HR prostate cancer, UHR & FHR, 5 year bRFS rates were 58.2%, 66.2%, and 69.2%, and 5 year DMFS rates were 78.4%, 81.2%, and 88.0%.

**Conclusion:**

Patients with multiple HR factors have worse outcome than patients with 1 HR factor. Future studies should account for this heterogeneity in HR prostate cancer.

## Background

Risk stratifying newly diagnosed prostate cancer aids physicians and patients to choose an optimal management approach. The most widely used classification system for prostate cancer was developed in 1998 [1] and this has been adapted by the National Comprehensive Cancer Network (NCCN) [2]. High-risk prostate cancer is defined by the 2015 NCCN guidelines as biopsy Gleason Score (bGS) of 8 to 10, or prostate-specific antigen (PSA) concentration of >20 ng/ml, or clinical stage T3a [2]. Patients with multiple intermediate risk factors (for example, a bGS of 7 and PSA of 10–20 ng/ml) may also be considered for management similar to patients with high-risk disease [2, 3]. This risk stratification scheme was originally based on biochemical outcomes of patients treated with standard doses of external beam radiation therapy (EBRT) in the order of 70 Gy or less [1].

A current standard of care for the management of high-risk disease is radiation dose escalation combined with androgen deprivation therapy (ADT). This combination has shown improvement in rates of biochemical failure and distant metastases compared to standard dose radiation alone [4–8]. It remains unclear whether the original high-risk definition based on biochemical outcomes remains applicable to patients treated in this manner for more clinically relevant endpoints of distant metastases and prostate cancer-specific mortality.

Although most high-risk prostate cancer patients fare well after curative therapy, a subgroup of patients still succumbs to their disease despite aggressive treatment. Therefore, there is a need to revisit our classification system and attempt to better stratify patients within this heterogeneous disease. The aim of our study was to sub-stratify high-risk prostate cancer patients treated with high-dose, image-guided, conformal radiation therapy and androgen deprivation into prognostic subgroups using combinations of accepted risk factors.

## Methods

Using prospectively gathered data from our six prostate cancer clinical trials (ICORG 97–01, ICORG 02–01, ICORG 05–04, ICORG 06–15, ICORG 06–16, ICORG 08–17,) we identified 547 patients who were treated with definitive EBRT (≥70Gy) at the St Luke’s Radiation Oncology Network from 1997 to 2013.

Routine workup included history and physical examination and digital rectal examination. Patients with distant or nodal metastatic disease as identified on bone scans or CT scans of the abdomen and pelvis were excluded. All patients were treated with either high-dose three-dimensional conformal radiotherapy or intensity-modulated RT (IMRT). Radiotherapy was delivered in accordance with trial protocol (e.g. for 05–03 and 06–15 it was as per institutional policy; for 06–16 and 08–17 the clinical target volume included the prostate and seminal vesicles, and for 97–01 and 02–01 the clinical target volume included the prostate and seminal vesicles, and the planning target volume encompassed the clinical target volume with a margin of 1 cm). The pelvic lymph nodes were not electively irradiated. No patient received a brachytherapy boost. All radiation treatment was delivered with image guidance per trial protocol.

Androgen deprivation therapy (ADT) was used in 98% of patients, with a luteinizing hormone-releasing hormone agonist given before and/or concurrently with and after EBRT. The use of an additional oral non-steroidal anti-androgen and the total duration of ADT were either prescribed by the trial protocol or left to the discretion of the treating physician. Patients were routinely followed with serum PSA testing at least every 6 months, and metastatic workup was initiated only if clinically indicated.

### Statistical analyses

Biochemical failure was calculated using the nadir PSA plus 2 ng/ml definition [9]. The percentage of biopsy cores positive for prostate cancer and the pre-treatment PSA velocity were not available for all patients and were not included in this analysis. BRFS (time to any event including biochemical relapse, irrespective of cause, except for any second primary cancers) and DMFS (time to lymph node/ bone/ distant soft tissue metastasis or death from any cause) were defined as the time from the start of RT to date of event or the date of last follow-up if there was no event.

The Kaplan-Meier method was used to estimate bRFS and DMFS [10]. The Cox proportional hazards model] was used to assess the impact of potential explanatory variables on survival times [11]. These included clinical stage (T1b- T2a, T2b-T2c and T3a), PSA (continuous), bGS (≤6, 7, 8–10), duration of ADT (continuous and stratified by ≤6 months vs. >6 months), and radiation dose (continuous). The log-rank test was used to compare differences in survival. All statistical tests were two-sided and assessed for significance at the 0.05 level. Statistical analyses were carried out using IBM® SPSS® statistical software version 22.

Four subgroups were defined: (1) two to three NCCN Intermediate risk factors (*n* = 104); (2) one NCCN High risk factor (*n* = 225); (3) two to three NCCN High risk factors (*n* = 92); (4) NCCN very high risk factor (*n* = 126). Subgroups (2 and 3) were then split into two dichotomous groups based on 5-year DMFS rates, either above or below the median DMFS rate for all patients, to define a favourable high-risk (FHR) cohort and an unfavourable high-risk (UHR) cohort of high-risk prostate cancer. These groups were then compared to the very high-risk group for bRFS and DMFS.

## Results

For the 547 patients, the median age was 68 years old (range, 46–83 years old), the median baseline PSA was 14.5 ng/ml (range, 0.6–263 ng/ ml), and 27% of patients had a bGS of 8 to 10. The median EBRT dose to the planning target volume was 74 Gy (range, 70–81 Gy) in 2Gy/ fraction biologically equivalent doses, and IMRT was used with 21% of all patients. Overall, 98% of men received ADT for a median duration of 8 months and a mean duration of 15 months (range, 2–72 months).

The median follow-up was 62.3 months (range, 0–183.7 months). For the entire study cohort, the 2-year bRFS rate was 87%, the 2-year and 5-year DMFS rates were 95% and 85%.

On univariate analysis for all patients, using Cox proportional hazards regression, statistically significant predictors of longer bRFS were T stage (T1b-T2a and T2b-T2c versus T3b-T4), PSA level at randomisation, duration of ADT, and radiation dose (Table 1). T3a stage did not predict for longer bRFS than T-stages T3b–T4. BGS (<7, 7, 8–10) was not a statistically significant predictor of bRFS but was retained in the multivariate model because of its clinical significance. A bGS <7 and =7 (vs. 8–10; *p* < .0005 and *p* = .010 respectively), PSA level (*p* < .0005), duration of ADT (continuous; *p* = .017), and radiation dose (*p* < .0005) were significant predictors for longer bRFS on multivariate analysis. T-stage did not remain significant in the presence of the other variables (*p* = .112).

**Table 1:** Univariate and multivariate analysis of risk factors for bRFS and DMFS

	Risk factor	Reference category	Univariate analysis			Multivariate analysis		
			HR	95% CI	p	HR	95% CI	p
bRFS								
	T-stage	T3b-T4			0.047			0.112
	T1b-T2a		0.64	0.43–0.94	0.025	0.653	0.44–0.98	0.038
	T2b-T2c		0.60	0.39–0.91	0.016	0.672	0.43–1.04	0.075
	T3a		0.76	0.53–1.09	0.134	0.91	0.63–1.30	0.608
	PSA		1.01	1.01–1.02	<.0005	1.015	1.01–1.02	<0.0005
	Gleason score	GS > 7			0.148			<0.0005
	< 7		0.68	0.46–1.00	0.053	0.349	0.23–0.53	<0.0005
	= 7		0.83	0.58–1.19	0.307	0.604	0.41–0.89	0.010
	ADT duration (continuous)		0.97	0.96–0.99	0.001	0.979	0.96–1.00	0.017
	Radiation dose		0.83	0.77–0.89	<0.0005	0.859	0.79–0.93	<0.0005
DMFS								
	T-stage	T3b-T4			0.204			0.272
	T1b-T2a		0.62	0.37–1.02	0.061	0.634	0.38–1.06	0.081
	T2b-T2c		0.65	0.38–1.11	0.117	0.695	0.40–1.22	0.204
	T3a		0.84	0.54–1.29	0.424	0.916	0.59–1.42	0.694
	PSA		1.01	1.00–1.02	0.004	1.009	1.00–1.01	0.011
	Gleason score	GS > 7			0.137			0.013
	< 7		0.61	0.37–0.99	0.047	0.482	0.29–0.79	0.004
	= 7		0.77	0.49–1.21	0.254	0.730	0.46–1.17	0.189
	ADT duration (continuous)		0.99	0.97–1.01	0.366			
	Radiation dose		0.86	0.78–0.95	0.004	0.853	0.77–0.95	0.003

Abbreviations: *bRFS* biochemical relapse-free survival, *CI* confidence interval, *DMFS* distant metastases-free survival

On univariate analysis, statistically significant predictors of longer DMFS were low PSA level, and higher radiation dose (Table 1). Shorter duration of ADT, T-stage (grouped), and bGS (grouped) were not statistically significant predictors of DMFS, but T-stage (grouped) and bGS (grouped) were retained in the multivariate model because of their clinical significance. A bGS <7 (vs. 8–10; *p* = .004), low PSA level (*p* = .011), and higher radiation dose (*p* = .003) were significant predictors for longer DMFS on multivariate analysis. T-stage (*p* = .272) was not a significant predictor of DMFS.

**Fig. 1:**
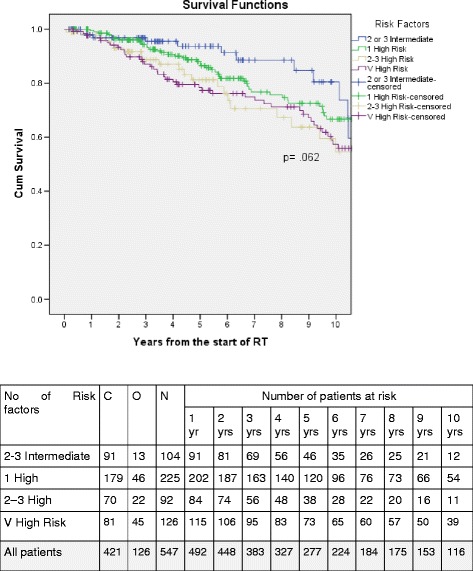
Kaplan-Meier curves of distant metastases-free survival by high-risk subgroup. Abbreviations: C = censored; O = observed; N = number

**Fig. 2:**
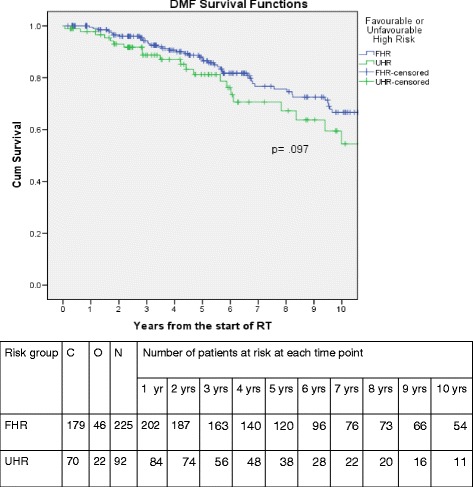
Kaplan-Meier curves of distant metastases-free survival by favourable versus unfavourable high risk groups.. Abbreviations: FHR = favourable high risk; UHR = unfavourable high risk; C: censored; O: observed; N: number

Kaplan-Meier curves were generated, and 5-year DMFS rates were calculated for each of the four subgroups (Fig. 1, Table 2). These patients were then split into two dichotomous groups (1) FHR: 1 high risk factor (*n* = 225); and (2) UHR (*n* = 92): 2–3 high risk factors. The patient and treatment characteristics of these high-risk cohorts are listed in Table 3. The median duration of ADT use was 8 and 13.5 respectively. The estimated 2-year rates of bRFS were 91.6% and 78.8%, respectively for FHR and UHR. The 5 year bRFS rates were 69.2%, 66.2% and 58.2%, respectively, for FHR, UHR and very-high risk groups. The 2-year rates of DMFS were 96.5% and 93.0%, respectively, for FHR and UHR (Table 4). The 5-year rates of DMFS were 88.0% and 81.2% respectively, for FHR and UHR. The estimated 10-year rates of DMFS were 66.7% and 54.5% respectively, for FHR and UHR. These results are graphically shown in Fig. 2. The estimated 5 and 10-year rates of DMFS for the very high-risk group were 78.4% and 57.4% respectively. On Cox proportional hazards regression analysis, There was a trend to shorter distant metastasis free survival for UHR than for FHR patients (*p* = 0.097). The median DMFS was not yet reached for the FHR group compared to 10.9 years for the UHR group. (Fig. 2) On Cox proportional hazards regression analysis, there was a trend towards a higher risk of biochemical relapse and distant metastasis for UHR than for FHR patients (Table 5). The estimated hazards or risks of biochemical relapse and distant metastasis increases by 1.4 and 1.5 times, respectively, for those with UHR compared to those with FHR.

**Table 2:** 5-year rates of DMFS by sub-group

Sub-group	Number of patients	5-year DMFS (%)	95% CI
All patients	421	87.8	84.1–91.5
2–3 Intermediate risk factors	104	93.7	88.2–99.2
1 High risk factor	225	88.0	83.1–92.9
2–3 High risk factors	92	81.2	71.6–90.8
V High Risk	126	78.4	70.8–86.0

Abbreviations: *CI* confidence interval, *DMFS* distant metastases-free survival

**Table 3:** Patient and treatment characteristics by high-risk cohort

Characteristic	All patients (*n* = 547)		FHR (*n* = 225)		UHR (*n* = 92)		vHigh Risk (*n* = 126)		FHR vs. UHR *p*-value
		%		%		%		%	
Age (years)									
Median	67.0		67.4		68.4		67.2		278
Range	46–83		47–83		46–80		48–81		
T Stage (no.)									<.0005
T1b–T2a	132	24	78	35	10	11	0	0	
T2b–T2c	121	22	51	23	10	11	0	0	
T3a	168	31	96	43	72	78	0	0	
T3b-T4	126	23	0	0	0	0	126	100	
PSA (μg/L)									<.0005
Median	14.5		14.1		22.5		14.9		
Mean	20.2		18.3		31.2		21.9		
Range	0.6–263		0.6–69		1.8–263		1.2–127		
PSA Group (no.)									<.0005
< 10 μg/L (no.)		28		33		17	37	29	
10–20 μg/L	151	38	74	34	16	10	44	35	
(no.)	209	34	76	33	9	73	45	36	
≥ 20 μg/L (no.)	187		75		67				
Gleason Score (No.)									<.0005
<7	147	27	80	36	11	12	50	40	
=7	250	46	91	40	24	26	37	29	
>7	150	27	54	24	57	62	39	31	
Radiation dose									
Median (Gy)	74.0		74.0		74.0		70.0		.082
Radiation technique									.123
3D–CRT	432	79	170	76	61	66	112	89	
IMRT	115	21	55	24	31	34	14	11	
Duration of ADT									
Median (months)	8.0		8.0		13.5		8.0		.102
Mean (months)	14.9		16.4		20.8		13.7		
Range (months)	0–72		0–53		4–72		0–68		
Duration of ADT (no)									
None (no.)	11	2	4	2	0	0	1	1	
1–6 months (no.)	181	32	69	31	24	26	44	35	
7–12 months (no.)	195	34	74	33	22	24	50	40	
13–24 months (no.)	10	2	3	1	4	4	2	2	
>24 months (no.)	150	29	75	33	42	46	29	23	

Abbreviations: *FHR* favourable high risk, *UHR* unfavourable high risk, *PSA* prostate-specific antigen, *ADT* androgen deprivation therapy, *3D–CRT* three dimensional conformal radiotherapy, *IMRT* intensity-modulated radiation therapy

**Table 4:** 2-year and 5-year rates of bRFS and DMFS

Sub-group	2-year rate (%)	95% CI	5-year rate (%)	95% CI
bRFS				
FHR	91.6	87.7–95.5	69.2	61.9–76.5
UHR	78.8	70.0–87.6	66.2	54.6–77.8
vHigh Risk	79.4	72.0–86.8	58.2	48.8–67.6
DMFS				
FHR	96.5	94.0–99.0	88.0	83.1–92.9
UHR	93.0	87.5–98.5	81.2	71.6–90.8
vHigh risk	93.2	88.7–97.7	78.4	70.8–86.0

Abbreviations: *bRFS* biochemical relapse-free survival, *CI* confidence interval, *DMFS* distant metastases-free survival

**Table 5:** Cox proportional hazards regression analysis by high-risk subgroup

End point	Risk-group	Reference category	HR	95% CI	*p*-value
bRFS	UHR	FHR	1.36	0.89–2.06	0.155
DMFS	UHR	FHR	1.54	0.92–2.57	0.100

Abbreviations: *FHR* favourable high risk, *UHR* unfavourable high risk, *bRFS* biochemical relapse-free survival, *DMFS* distant metastases-free survival, *HR* hazard ratio, *CI* confidence interval

## Discussion

Numerous definitions of high-risk prostate cancer exist [1, 12–16]. Lack of clarity and consensus on a single precise definition represents a potential barrier for patient-specific counselling and comparative assessment of different treatment modalities.

The optimal treatment for men with high-risk disease remains debatable, although treatment options include surgery, radiation, and/or ADT [4–8, 17–21]. Currently regardless of the treatment used, there is significant heterogeneity in outcomes from high-risk prostate cancer [18–25]. One potential way to overcome this heterogenity, is by defining high risk by adding several high-risk factors.

This study sought to report on the outcomes of patients with high-risk prostate cancer treated on prospective clinical trials. Numerous randomized trials have established the combination of EBRT and ADT as one of the standards of care for treating high- risk prostate cancer [4–6, 26, 27]. The optimal duration of hormone therapy remains unknown. However, many trials have shown superior outcomes with a longer duration of ADT [6, 28]. The majority of these earlier clinical trials establishing the value of ADT, used suboptimal radiation doses, typically in the order of 70 Gy [8, 19–21]. Currently, delivery of high-dose EBRT in high-risk prostate cancer is standard of care [4–8].

In our analysis we included patients with multiple intermediate factors as per NCCN criteria, these could be managed as high-risk disease [2]. We felt that they would also act as a good comparative arm for patients with high-risk and very high-risk factors. We sought to incorporate several high-risk factors to help potentially distinguish distinct subgroups within high-risk prostate cancer. We found that a high GS of 8 to 10, higher PSA level and lower radiation dose were significant predictors of shorter DMFS on multivariate analyses. T stage at diagnosis did not predict distant metastasis-free survival. Looking at bRFS high GS of 8 to 10, higher PSA level, lower radiation dose & shorter duration of ADT were significant predictors of shorter bRFS on multivariate analyses.

By doing this, we demonstrated the marked heterogeneity in this disease. Our data suggest that there may be two dichotomous subgroups of high-risk patients. One group with one high-risk factor, whose 5 year DMFS outcomes are similar to the intermediate risk prostate cancer group, may have excellent outcomes with a short duration of ADT and high-dose radiotherapy limited to the prostate and seminal vesicles. On the other hand, patients in the unfavourable high-risk subgroup, patients with multiple high-risk features had a poor prognosis despite longer duration of ADT, with risk of metastasis approaching 20% at 5 years. Our results are consistent to outcomes from John Hopkins who reported similar results in a surgical cohort of patients [29] and in patients treated with ADT and high-dose radiotherapy [30]. They identified similar subpopulations of patients with NCCN high-risk men who experienced inferior outcomes following definitive radiation and long-term androgen deprivation therapy (ADT). They noted that the 10-year risk of distant metastasis (DM) of 35%, for patients with unfavourable high risk disease. This was far worse than NCCN high-risk men whose 10 year risk of distant metastasis (DM) was 13%. Our study adds to this body of evidence with much worse 10-year risk of distant metastasis (DM) in the UHR cohort when compared to the FHR cohort.

Consistent with other reports, a high GS is the strongest driver for bRFS and DMFS (Table 1), particularly when combined with other high-risk factors [3, 21]. Patients with multiple high-risk factors could be ideal candidates for clinical trials investigating more aggressive treatment strategies.

The use of a longer duration of ADT, when given with high-dose radiation, does improve bRFS on multivariate analyses but does not predict DMFS. The median duration of ADT in this study overall was 8 months. Our findings showing marginal benefit to long term hormones in high risk prostate cancer are consistent with prior publications that have sought to reclassify high-risk prostate cancer [22, 23]. This analysis validates the importance of aggressive local treatment as escalated radiation doses reduce the risk of distant failure consistent with other series [24, 25]. This may have implications for future trial design. Given the lower rate of distant metastasis in the favourable cohort of high-risk prostate cancer, these patients could conceivably be managed in a similar way to unfavourable intermediate risk prostate cancer. Future studies might focus on the unfavourable high-risk prostate cancer group in the design of clinical trials, where a bigger benefit is likely to be seen.

We are aware of the existence of several high-risk definitions. None discriminates accurately between patients who are likely to do well, and patients do worse. We specifically chose to examine the current NCCN high-risk classification because it is widely used and simple in design. We recognize that several clinical factors must be considered when identifying those with an unfavorable prognosis and encourage future studies to incorporate as many predictive markers as possible to better define the high-risk population.

Our study has successfully demonstrated an ability to stratify high-risk prostate cancer into a favorable and unfavourable subgroup. Our favourable subgroup has 5 year DMFS outcomes comparable to unfavourable intermediate risk prostate cancer, and our unfavourable high-risk group have outcomes more in line with very high-risk disease, a cohort which received lower radiation dose and a shorter duration of ADT. Our findings might help to direct future clinical trial design and may help personalize care for individual patients. There are a number of current studies with preliminary results which are specifically looking patients with high risk disease to see if the addition of brachytherapy or enzalutamide may optimize disease control [31, 32].

These results highlight the heterogeneity within high-risk prostate cancer. This is one of the first series from Europe in patients with high-risk prostate cancer treated with radiotherapy that has sought to sub-classify high-risk disease. Other series have been in high-risk prostate cancer patients managed surgically. Three studies have shown that the presence of more high-risk features may predict for worse cancer-specific outcomes among patients with high-risk prostate cancer treated with radical prostatectomy suggesting that there is an additive effect from each individual risk factors [17, 18]. Some North American studies looking at patients with high-risk disease treated with ADT and RT, one of which validated their data against the SEER database, have also managed to sub-classify high-risk disease [22, 23]. However a significant strength of our study was that we used data from six clinical trials which was prospectively gathered.

There are limitations to our study. First, our results are based on a secondary analysis of the combined data from 6 prostate cancer clinical trials and should be interpreted cautiously. Second, our outcomes in the unfavourable high-risk group are similar to patients in the very high-risk group. However this is in an era before the use of MRI staging, so there may be significant stage migration in a more modern cohort. Finally, picking 5 year DMFS rates as the cut-off may be arbitrary or may mask other salvage treatments.

We await further prospective evaluations for high-risk patients, and encourage future studies to consider the wide heterogeneity in this cohort. Several biomarkers are under investigation as predictive tools but none is clinically available yet, and it remains to be seen how these will translate into the management of patients with prostate cancer [33]. Novel radiographic techniques and molecular markers which help predict relapse following treatment are needed to help us more accurately direct individualised treatment. Until such markers exist, additional clinical information from prostate biopsy, pretreatment PSA velocity, and radiographic findings from endorectal MRI may help to predict better and enable more accurate discrimination between the different risk groups.

## Conclusion

In summary, high-risk prostate cancer is a widely heterogeneous disease. In patients treated with high dose radiotherapy and ADT, bGS, PSA level, duration of ADT, and radiation dose were significant predictors for bRFS after adjustment for the effects of the other covariates. With the exception of duration of ADT, these variables were also predictive for DMFS. We identified an unfavorable group of high-risk prostate cancer patients, similar to those with very high-risk disease, with significantly shorter times to distant metastases than the favourable high risk group. We encourage future clinical trials to consider the marked heterogeneity in this disease.

## References

[1] D’Amico AV, et al. Biochemical outcome after radical prostatectomy, external beam radiation therapy, or interstitial radiation therapy for clinically localized prostate cancer. JAMA. 1998;280(11):969–74. 10.1001/jama.280.11.969 9749478

[2] Prostate O, Washington PA (2015). National Comprehensive Cancer Network Clinical Practice Guidelines in NCCN, fort.

[3] Tsai HK (2006). Cancer-specific mortality after radiation therapy with short-course hormonal therapy or radical prostatectomy in men with localized, intermediate-risk to high-risk prostate cancer. Cancer.

[4] Bolla M (2002). Long-term results with immediate androgen suppression and external irradiation in patients with locally advanced prostate cancer (an EORTC study): a phase III randomised trial. Lancet (London, England).

[5] Pilepich MV (2005). Androgen suppression adjuvant to definitive radiotherapy in prostate carcinoma--long-term results of phase III RTOG 85-31. Int J Radiat Oncol Biol Phys.

[6] Horwitz EM (2008). Ten-year follow-up of radiation therapy oncology group protocol 92-02: a phase III trial of the duration of elective androgen deprivation in locally advanced prostate cancer. J Cli Oncol Off J Am Soc Clin Oncol.

[7] Kuban DA (2008). Long-term results of the M. D. Anderson randomized dose-escalation trial for prostate cancer. Int J Radiat Oncol Biol Physi.

[8] Zelefsky MJ (2008). Long-term results of conformal radiotherapy for prostate cancer: impact of dose escalation on biochemical tumor control and distant metastases-free survival outcomes. Int J Radiat Oncol Biol Phys.

[9] Roach M (2006). Defining biochemical failure following radiotherapy with or without hormonal therapy in men with clinically localized prostate cancer: recommendations of the RTOG-ASTRO phoenix consensus conference. Int J Radiat Oncol Biol Phys.

[10] Kaplan EL, Meier P, Am J (1958). Nonparametric estimation from incomplete observations. Stat Assoc.

[11] Cox DR, Roy J (1972). Regression models and life tables. Stats Soc.

[12] Thompson I (2007). Guideline for the management of clinically localized prostate cancer: 2007 update. J Urol.

[13] Roach M (2000). Four prognostic groups predict long-term survival from prostate cancer following radiotherapy alone on radiation therapy oncology group clinical trials. Int J Radiat Oncol Biol Phys.

[14] Roach M (2006). Defining high risk prostate cancer with risk groups and nomograms: implications for designing clinical trials. J Urol.

[15] Huang J (2012). Percentage of positive biopsy cores: a better risk stratification model for prostate cancer?. Int J Radiat Oncol Biol Phys.

[16] Cooperberg MR (2005). The University of California, san Francisco cancer of the prostate risk assessment score: a straightforward and reliable preoperative predictor of disease recurrence after radical prostatectomy. J Urol.

[17] Walz J (2011). Pathological results and rates of treatment failure in high-risk prostate cancer patients after radical prostatectomy. BJU Int.

[18] Spahn M (2010). Outcome predictors of radical prostatectomy in patients with prostate-specific antigen greater than 20 ng/ml: a European multi-institutional study of 712 patients. Eur Urol.

[19] Zietman AL (2005). Comparison of conventional-dose vs high-dose conformal radiation therapy in clinically localized adenocarcinoma of the prostate: a randomized controlled trial. JAMA.

[20] Al-Mamgani A (2008). Update of Dutch multicenter dose-escalation trial of radiotherapy for localized prostate cancer. Int J Radiat Oncol Biol Phys.

[21] Nanda A (2009). Gleason pattern 5 prostate cancer: further stratification of patients with high-risk disease and implications for future randomized trials. Int J Radiat Oncol Biol Phys.

[22] Tendulkar RD (2012). Redefining high-risk prostate cancer based on distant metastases and mortality after high-dose radiotherapy with androgen deprivation therapy. Int J Radiat Oncol Biol Phys.

[23] Muralidhar V (2015). Definition and validation of “favorable high-risk prostate cancer”: implications for personalizing treatment of radiation-managed patients. Int Oncol Biol Phys 93.

[24] Cahlon O (2008). Ultra-high dose (86.4 Gy) IMRT for localized prostate cancer: toxicity and biochemical outcomes. Int J Radiat Oncol Biol Phys.

[25] Liauw SL (2010). Dose-escalated radiotherapy for high-risk prostate cancer: outcomes in modern era with short-term androgen deprivation therapy. Int J Radiat Oncol Biol Phys.

[26] Amico A V D’, Manola J, Loffredo M (2004). 6-month androgen suppression plus radiation therapy vs radiation therapy alone for patients with clinically localized prostate cancer a randomized controlled trial. JAMA.

[27] Bolla M (2009). Duration of androgen suppression in the treatment of prostate cancer. The New England J Med.

[28] Dearnaley DP (2007). Escalated-dose versus standard-dose conformal radiotherapy in prostate cancer: first results from the MRC RT01 randomised controlled trial. Lancet Oncol.

[29] Sundi (2014). Very-high-risk localized prostate cancer: definition and outcomes. Prostate Cancer Prostatic Dis.

[30] Narang AK, et al. Very high-risk localized prostate cancer: outcomes following definitive radiation. Int J Radiat Oncol Biol Phys. 2016;94(2):254–62. 10.1016/j.ijrobp.2015.10.056 PMC5065713 26853334

[31] Morris MJ (2015). Radiographic progression-free survival as a response biomarker in metastatic castration-resistant prostate cancer: COU-AA-302 results. J Clin Oncol: Official J Am Soc Clin Oncol.

[32] Williams SG, et al. Randomised phase 3 trial of enzalutamide in androgen deprivation therapy (ADT) with radiation therapy for clinically localised high-risk or node-positive prostate cancer: ENZARAD (ANZUP 1303). 2016;TPS5086-TPS5086.

[33] Roach M, Waldman F, Pollack A (2009). Predictive models in external beam radiotherapy for clinically localized prostate cancer. Cancer.

